# Consumption of Sugar-Sweetened Beverages Among US Adults in 6 States: Behavioral Risk Factor Surveillance System, 2011

**DOI:** 10.5888/pcd11.130304

**Published:** 2014-04-24

**Authors:** Sohyun Park, Liping Pan, Bettylou Sherry, Heidi M. Blanck

**Affiliations:** Author Affiliations: Liping Pan, Bettylou Sherry, Heidi M. Blanck, Centers for Disease Control and Prevention, Atlanta, Georgia.

## Abstract

**Introduction:**

Sugar-sweetened beverage (SSB) intake is linked to weight gain. Our objective was to examine state-specific SSB intake and behavioral characteristics associated with SSB intake.

**Methods:**

We used data from the 2011 Behavioral Risk Factor Surveillance System for 38,978 adults aged 18 years or older from 6 states: Delaware, Hawaii, Iowa, Minnesota, New Jersey, and Wisconsin. Multivariate logistic regression was used to estimate adjusted odds ratios for characteristics associated with SSB intake from regular soda and fruit drinks.

**Results:**

Overall, 23.9% of adults drank SSBs at least once a day. Odds of drinking SSBs 1 or more times per day were significantly greater among younger adults; males; non-Hispanic blacks; adults with lower education; low-income adults or adults with missing income data; adults living in Delaware, Iowa, and Wisconsin versus those living in Minnesota; adults with fruit intake of less than 1 time a day versus 1 or more times a day; adults who were physically inactive versus highly active adults; and current smokers versus nonsmokers. Odds for drinking SSBs 1 or more times per day were significantly lower among adults with 100% fruit juice intake of less than 1 time per day versus 1 or more times per day and among adults who drank alcohol versus those who did not drink alcohol.

**Conclusion:**

SSB intake varied by states and certain sociodemographic and behavioral characteristics. States can use findings from this study to tailor efforts to decrease SSB intake and to encourage consumption of more healthful beverages (eg, water) among their high-risk populations.

## Introduction

Sugar-sweetened beverages (SSBs) contribute significant number of calories to diets of US adults (1). The 2010 Dietary Guidelines for Americans define SSBs as “liquids that are sweetened with various forms of sugars that add calories. These beverages include, but are not limited to, soda, fruit-ades and fruit drinks, and sports and energy drinks” (2). In the National Health and Nutrition Examination Survey (NHANES) 2005–2008, about half of Americans drank SSBs on any given day (3). SSB intake in adults is associated with obesity (4–6); type 2 diabetes (6–8); increased risk for cardiovascular disease (6,9–11), nonalcoholic fatty liver disease (12), kidney disease (13), and gout (14); and decreased diet quality (15).

Several studies have examined sociodemographic characteristics associated with SSB intake among a nationally representative sample of US adults (3,16–19). State-specific information on SSB intake is limited, and only a few studies have examined associations between behavioral factors and SSB intake among US adults. For example, a study reported that obese adults and those who were not trying to lose weight had higher mean calorie intake from SSBs (17). Another study found no differences in SSB intake by physical activity and smoking among adults aged 20 to 39 years (19). Identifying behavioral factors and state-specific information associated with SSB intake can enable states to target intervention efforts to decrease SSB intake among high-risk populations. Additionally, the Centers for Disease Control and Prevention (CDC) has selected reducing SSB intake as a target behavior for preventing and controlling obesity and supports states in working on state-level data to prioritize subgroups that may need more targeted interventions for SSB reduction. State-based surveillance data are needed to document baseline and postintervention SSB consumption to monitor the impact of CDC funding of state-based interventions to prevent and control obesity.

This study provides a summary of the first available standardized data for the 6 states that used the standardized Behavioral Risk Factor Surveillance System (BRFSS) Optional Module for assessing SSB intake: Delaware, Hawaii, Iowa, Minnesota, New Jersey, and Wisconsin. Our objective was to describe SSB intake (regular soda/fruit drinks) in adults by using a state-specific data source and to examine the associations between SSB intake and sociodemographic and behavioral characteristics.

## Methods

### Sample and survey administration

This cross-sectional study was based on 2011 BRFSS data. BRFSS is a state-based, telephone interview survey conducted annually by CDC and state health departments and is designed to assess a range of respondents’ health conditions and behaviors related to public health issues. It uses a multistage cluster sampling design with random-digit dialing (including landline and cellular telephones) to select a sample that represents the civilian, noninstitutionalized adult population in each of the 50 states, the District of Columbia, and 3 US territories. Poststratification weights are used to adjust for nonresponse, noncoverage, and uneven selection of the population to generate demographic distributions that closely match the state population. Detailed information on validity of measures and survey data is available elsewhere (20,21). Every year, several Optional Modules are available on BRFSS, and states decide whether to include these as part of their survey. A standardized Optional Module for assessing soda and fruit drink intake as surrogates for total SSB intake was made available on the 2011 BRFSS, and the 6 states included in this study used this module. Although this SSB Optional Module was offered to states in 2012, we used 2011 data to provide information for the initial year of module use and to include key behavioral variables (eg, fruit and vegetable intake, physical activity) that were not collected in 2012. A total of 43,157 adults aged 18 years or older completed the SSB Optional Module. For this analysis, 4,179 adults with missing data on regular soda (soda sweetened with various forms of sugars) or fruit drink intake were excluded, resulting in a final analytic sample of 38,978 adults. In comparing the analytic sample with adults who were excluded from the study because of missing outcome data, the analytic sample had a slightly lower proportion of adults aged 70 years or older and a higher proportion of non-Hispanic whites; the sample had no difference in sex. Additionally, unknown values and missing data for exposure variables ranged from 0.2% to 5.5% (the latter for race/ethnicity) and were excluded from analyses when the variable was used.

### Outcome variable

The outcome measure was SSB intake. Respondents were asked “About how often do you drink regular soda or pop that contains sugar? Do not include diet soda or diet pop,” and “About how often do you drink sweetened fruit drinks, such as Kool-Aid, cranberry juice, and lemonade? Include fruit drinks you made at home and added sugar to.” For each question, respondents reported number of times per day, per week, or per month they consumed these beverages. Weekly or monthly intake was converted to daily intake (dividing weekly intake by 7 and monthly intake by 30). To calculate total SSB intake frequency, the frequency of consumption of regular soda and fruit drinks was summed. Of note, although past month SSB intake was converted to daily consumption, this may not capture day-to-day variability in consumption. For χ^2^ tests, 4 mutually exclusive SSB intake categories were created for per-day consumption (0, >0 to <1, 1 to <2, and ≥2 times). The cutpoint of 1 time per day was chosen to define daily intake, and the cutpoint of 2 times per day was based on the estimated 85th percentile of energy intake from SSB on a given day (about two 12-oz cans of soda) among Americans (3,22).

### State and sociodemographic and behavioral variables

Mutually exclusive response categories were created for exposure variables. Sociodemographic variables included were age (18–29 y, 30–39 y, 40–49 y, 50–59 y, 60–69 y, or ≥70 y), sex, race/ethnicity (non-Hispanic white, non-Hispanic black, Hispanic, or other), education level (<high school, high school graduate, some college, or college graduate), marital status (married, single, divorced/separated/widowed), and annual household income (<$25,000, $25,000–$49,999, $50,000–$74,999, ≥$75,000, or don’t know/refused/missing). Because a relatively large proportion (~14%) of adults had “don’t know/refused/missing” responses for annual household income, we did not exclude these individuals from our analyses. Instead, we created an additional category for these adults and included them as such in our analyses.

We selected behavioral variables for study on the basis of a literature review (15,17,19). Furthermore, the cutoffs for the consumption of fruits (<1 time/d or ≥1 time/d), 100% fruit juice (<1 time/d or ≥1 time/d), and vegetables (<3 times/d or ≥3 times/d) were based on a previous study (23). Leisure-time physical activity was categorized as highly active (>300 min/wk), active (150–300 min/wk physical activity), insufficiently active (11–149 min/wk physical activity), and inactive (no physical activity). Smoking status was categorized as nonsmoker, former smoker, or current smoker. Alcohol drinking was categorized as no drinking, any drinking (≥1 drink of any alcoholic beverage during the past month but not heavy drinking), and heavy drinking (>2 drinks/d for men and >1 drink/d for women). Body mass index (BMI) was calculated from self-reported weight and height (kg/m^2^), and weight status was categorized as underweight (BMI <18.5), normal weight (BMI 18.5–24.9), overweight (BMI 25.0–29.9), or obese (BMI ≥30) (24).

### Statistical analysis

We used χ^2^ tests to examine the unadjusted bivariate relationship between frequency of SSB intake and the sociodemographic and behavioral variables described above. The cutoff for statistical significance was *P* < .05. Multivariable logistic regression was used to estimate adjusted odds ratios and 95% confidence intervals for associations between the likelihood of drinking SSB 1 or more times per day and state and sociodemographic and behavioral characteristics. Data were pooled into 1 model with state as a variable in that model (n = 32,374 adults). The age, sex, and race/ethnicity distributions of the respondents included in the model were similar to those in the overall analytic sample (n = 38,978). The sample weight variable was applied to all analyses to provide valid estimates for the civilian noninstitutionalized adult population in each state. All statistical analyses were performed using SAS-Callable SUDAAN version 9.3 (SAS Institute, Cary, North Carolina) and incorporated appropriate procedures to account for the complex sample design.

## Results

Only 30% of adults reported never drinking SSBs (per day, week, or month). Overall, 70% reported drinking any SSB per day; 23.9% reported drinking SSBs at least once a day, and 10.7% reported doing so at least twice a day. By state, the prevalence of SSB intake 2 or more times per day was 14.4% for Iowa, 13.0% for Wisconsin, 10.9% for Delaware, 10.4% for Hawaii, 9.7% for Minnesota, and 8.5% for New Jersey (Table 1). SSB consumption varied by all sociodemographic and behavioral characteristics studied (χ^2^ tests, *P* < .05 for all variables). The proportion of adults drinking SSBs 2 or more times per day was highest among adults in Iowa, young adults, men, non-Hispanic blacks, adults with a less than high school education, single adults, and adults in the lowest-income category (Table 1). Regarding behavioral characteristics, the proportion of adults drinking SSBs 2 or more times per day was highest among adults consuming fruits less than once a day, 100% fruit juice less than once a day, and vegetables less than 3 times per day and among physically inactive adults, current smokers, adults who did not drink alcohol, and obese adults (Table 2).

**Table 1:** Prevalence of SSB^a^ Intake and Sociodemographic Characteristics of the Study Population and Their Associations with SSB Intake Among Adults in 6 States, Behavioral Risk Factor Surveillance System, 2011

Characteristic	Total	Sugar-Sweetened Beverage Intake				*P* Value^c^
	n (%)^b^	0 Times/Day, % (SE)	>0 to <1 Time/Day, % (SE)	1 to <2 Times/Day, % (SE)	≥2 Times/Day, % (SE)	
**Total sample (N = 38,978)**		**30.2 (0.5)**	**45.9 (0.6)**	**13.2 (0.4)**	**10.7 (0.4)**	**NA**
**States (n = 38,978)**						
Delaware	3,971 (3.9)	29.1 (1.1)	44.2 (1.3)	15.8 (1.0)	10.9 (0.9)	<.001
Hawaii	7,162 (5.8)	30.5 (0.8)	47.0 (0.9)	12.1 (0.6)	10.4 (0.6)	
Iowa	5,363 (12.3)	28.0 (0.8)	41.4 (1.0)	16.2 (0.9)	14.4 (0.9)	
Minnesota	13,804 (21.4)	29.8 (0.5)	48.0 (0.6)	12.6 (0.4)	9.7 (0.4)	
New Jersey	4,140 (34.5)	33.4 (1.1)	45.7 (1.3)	12.3 (0.9)	8.5 (0.8)	
Wisconsin	4,538 (22.1)	26.8 (0.9)	46.7 (1.2)	13.5 (0.9)	13.0 (0.8)	
**Age, y (n = 38,614)^d^**						
18–29	3,070 (19.3)	11.9 (1.2)	52.4 (1.8)	18.9 (1.4)	16.9 (1.2)	<.001
30–39	4,325 (17.0)	19.6 (1.0)	49.2 (1.5)	14.9 (1.1)	16.3 (1.2)	
40–49	6,241 (19.0)	30.2 (1.1)	46.7 (1.2)	11.8 (0.8)	11.3 (0.8)	
50–59	8,650 (19.0)	38.3 (0.9)	44.3 (1.0)	10.9 (0.6)	6.5 (0.5)	
60–69	8,081 (13.3)	42.5 (1.1)	41.0 (1.2)	10.6 (0.8)	5.9 (0.5)	
≥70	8,247 (12.5)	46.6 (1.0)	38.3 (1.0)	10.7 (0.7)	4.3 (0.4)	
**Sex (n = 38,978)^d^**						
Male	15,956 (49.1)	23.3 (0.6)	47.7 (0.9)	15.8 (0.7)	13.1 (0.6)	<.001
Female	23,022 (50.9)	36.8 (0.6)	44.1 (0.7)	10.7 (0.4)	8.4 (0.4)	
**Race/ethnicity (n = 36,849)^d^**						
White, non-Hispanic	29,907 (77.1)	32.5 (0.5)	45.0 (0.6)	12.8 (0.4)	9.7 (0.4)	<.001
Black, non-Hispanic	1,893 (6.9)	18.9 (2.3)	46.6 (2.7)	17.9 (2.0)	16.6 (2.1)	
Hispanic	1,483 (8.9)	21.4 (2.0)	47.7 (2.7)	14.9 (1.7)	15.9 (2.2)	
Other, non-Hispanic	3,566 (7.1)	26.8 (1.6)	54.2 (2.0)	12.1 (1.5)	6.9 (0.9)	
**Education level (n = 38,907)^d^**						
<High school	2,071 (10.8)	23.9 (1.6)	42.4 (2.1)	18.3 (1.5)	15.4 (1.6)	<.001
High school graduate	11,091 (30.7)	27.9 (0.8)	41.4 (1.0)	15.6 (0.8)	15.1 (0.8)	
Some college	11,007 (30.3)	29.2 (0.8)	47.3 (1.0)	12.8 (0.7)	10.7 (0.6)	
College graduate	14,738 (28.2)	36.0 (0.8)	50.6 (0.9)	9.2 (0.5)	4.2 (0.3)	
**Marital status (n = 38,806)^d^**						
Married	21,774 (53.5)	34.2 (0.6)	46.7 (0.6)	11.4 (0.4)	7.7 (0.4)	<.001
Single	6,774 (29.0)	17.2 (0.9)	48.6 (1.4)	17.1 (1.1)	17.0 (1.0)	
Divorced/separated/widowed	10,258 (17.6)	39.3 (0.9)	38.8 (0.9)	12.4 (0.6)	9.5 (0.6)	
**Annual household income, $ (n = 38,978)**						
<25,000	8,048 (20.9)	24.6 (0.9)	43.0 (1.3)	15.8 (1.0)	16.6 (1.0)	<.001
25,000–49,999	9,523 (22.4)	29.2 (0.9)	45.1 (1.0)	15.0 (0.8)	10.8 (0.6)	
50,000–74,999	5,963 (14.2)	29.4 (1.1)	50.3 (1.3)	11.4 (0.8)	8.9 (0.9)	
≥75,000	10,653 (28.3)	34.3 (0.9)	49.6 (1.0)	9.7 (0.7)	6.4 (0.5)	
Don’t know/refused/missing	4,791 (14.2)	32.3 (1.5)	39.7 (1.7)	15.6 (1.5)	12.4 (1.3)	

Abbreviations: SSB, sugar-sweetened beverage; SE, standard error; NA, not applicable.

a SSBs include nondiet soda and fruit drinks that are not 100% juice.

b Unweighted sample size and weighted percentage. Weighted percentages may not add to 100 because of rounding.

c χ^2^ tests were used for each variable to examine differences across categories.

d Sample size for age, race/ethnicity, education level, and marital status may not add up to total because of missing data.

**Table 2:** Prevalence of Behavioral Characteristics and Their Association with Sugar-Sweetened Beverage^a^ Intake Among Adults in 6 States,^b^ Behavioral Risk Factor Surveillance System, 2011

Characteristic	Sugar-Sweetened Beverage Intake					
	All, N (%)^c^	0 Times/Day, % (SE)	>0 to <1 Time/Day, % (SE)	1 to <2 Times/Day, % (SE)	≥2 Times/Day, % (SE)	*P* Value d
**Fruit intake (n = 38,101)**						<.001
<1 time/d	17,011 (48.9)	23.9 (0.6)	46.6 (0.8)	15.9 (0.7)	13.6 (0.6)	
≥1 time/d	21,090 (51.1)	36.0 (0.7)	45.6 (0.8)	10.7 (0.5)	7.7 (0.5)	
**100% fruit juice intake (n = 38,101)**						
<1 time/d	28,706 (76.1)	30.4 (0.5)	46.1 (0.6)	12.6 (0.4)	10.9 (0.4)	.048
≥1 time/d	9,395 (23.9)	29.1 (1.0)	46.0 (1.2)	15.2 (0.9)	9.7 (0.8)	
**Vegetable intake (n = 37,874)**						
<3 times/d	31,933 (86.8)	28.0 (0.5)	47.1 (0.6)	13.9 (0.4)	11.0 (0.4)	<.001
≥3 times/d	5,941 (13.2)	42.5 (1.4)	40.0 (1.4)	8.2 (0.7)	9.3 (1.0)	
**Leisure-time physical activity (n = 37,789)**						
Highly active	13,572 (33.1)	33.0 (0.8)	46.2 (1.0)	12.3 (0.7)	8.4 (0.6)	<.001
Active	7,420 (19.9)	30.0 (1.0)	49.9 (1.2)	12.2 (0.9)	7.9 (0.7)	
Insufficiently active	7,238 (20.5)	26.0 (1.0)	49.3 (1.2)	13.6 (1.0)	11.1 (0.8)	
Inactive	9,559 (26.6)	29.1 (0.9)	40.9 (1.1)	14.7 (0.8)	15.3 (0.9)	
**Smoking status (n = 38,815)**						
Nonsmokers	20,855 (55.4)	29.1 (0.6)	49.6 (0.8)	12.8 (0.5)	8.5 (0.5)	<.001
Former smokers	12,067 (26.1)	39.3 (0.9)	42.8 (0.9)	11.1 (0.7)	6.8 (0.5)	
Current smokers	5,893 (18.5)	20.4 (0.9)	39.1 (1.3)	17.6 (1.1)	22.9 (1.2)	
**Alcohol drinking (n = 38,781)**						
No drinking	15,969 (38.0)	30.8 (0.7)	42.8 (0.9)	14.2 (0.7)	12.2 (0.6)	<.001
Any drinking	20,199 (54.7)	29.7 (0.6)	48.4 (0.8)	12.6 (0.6)	9.4 (0.5)	
Heavy drinking^e^	2,613 (7.3)	30.4 (1.9)	44.4 (1.9)	13.1 (1.3)	12.1 (1.3)	
**Weight status (n = 37,065)**						
Underweight (BMI <18.5 kg/m^2^)	649 (1.7)	23.5 (3.0)	50.7 (4.9)	14.4 (2.6)	11.5 (2.6)	.007
Normal weight (BMI 18.5–24.9 kg/m^2^)	13,139 (35.8)	28.1 (0.8)	48.2 (1.0)	12.9 (0.7)	10.7 (0.7)	
Overweight (BMI 25–29.9 kg/m^2^)	13,521 (35.9)	30.5 (0.8)	46.5 (0.9)	13.5 (0.7)	9.5 (0.5)	
Obese (BMI ≥30 kg/m^2^)	9,756 (26.6)	31.4 (0.9)	42.4 (1.1)	13.7 (0.8)	12.5 (0.9)	

Abbreviations: SE, standard error; BMI, body mass index.

a Sugar-sweetened beverages include nondiet soda and fruit drinks that are not 100% juice.

b Delaware, Hawaii, Iowa, Minnesota, New Jersey, and Wisconsin.

c Percentages are weighted and may not total 100% because of rounding.

d χ^2^ tests were used for each variable to examine differences across categories.

e More than 2 drinks per day for men and more than 1 drink per day for women.

Multivariable logistic regression results showed that odds for drinking SSBs 1 or more times per day were significantly higher among adults in Delaware, Iowa, and Wisconsin versus adults in Minnesota; among younger adults than among those aged 70 or older; among men than among women; among non-Hispanic blacks than among non-Hispanic whites; among adults with lower education levels versus those who were college graduates; among adults with lower household income or with “missing/don’t know/refused” income data than among those with an income of $75,000 or more; among adults who consumed fruit less than once a day than among those who consumed fruit 1 or more times per day; among adults who were physically inactive than among those who were highly active; and among current smokers than among nonsmokers. In contrast, odds for drinking SSBs 1 or more time per day were significantly lower among adults consuming 100% fruit juice less than once per day than among those consuming 100% fruit juice 1 or more times per day and among those with any or heavy alcohol drinking than among nondrinkers (Table 3).

**Table 3:** Prevalence and Adjusted Odds Ratios of Consuming Sugar-Sweetened Beverages^a^ 1 or more Times Per Day Among Adults in 6 States, by Selected Characteristics, Behavioral Risk Factor Surveillance System, 2011

Characteristic	Sugar-Sweetened Beverage Intake ≥1 Time/Day^b^	
	% (SE)	Adjusted OR^c^ (95% CI)
**State**		
Delaware	26.7 (1.2)	1.26 (1.07–1.48)
Hawaii	22.5 (0.8)	1.18 (0.97–1.44)
Iowa	30.6 (1.1)	1.65 (1.44–1.89)
Minnesota	22.2 (0.6)	1 [Reference]
New Jersey	20.8 (1.1)	0.90 (0.75–1.07)
Wisconsin	26.5 (1.1)	1.29 (1.12–1.49)
**Age, y**		
18–29	35.8 (1.7)	3.37 (2.56–4.43)
30–39	31.2 (1.4)	3.35 (2.68–4.19)
40–49	23.1 (1.0)	2.15 (1.74–2.67)
50–59	17.4 (0.7)	1.37 (1.12–1.67)
60–69	16.5 (0.9)	1.15 (0.93–1.42)
≥70	15.1 (0.8)	1 [Reference]
**Sex**		
Male	28.9 (0.8)	1.81 (1.59–2.05)
Female	19.1 (0.6)	1 [Reference]
**Race/ethnicity**		
White, non-Hispanic	22.5 (0.5)	1 [Reference]
Black, non-Hispanic	34.5 (2.6)	1.41 (1.08–1.83)
Hispanic	30.9 (2.5)	1.06 (0.79–1.41)
Other, non-Hispanic	19.0 (1.7)	0.79 (0.60–1.04)
**Education level**		
<High school	33.7 (2.0)	1.99 (1.54–2.59)
High school graduate	30.7 (1.0)	2.03 (1.71–2.41)
Some college	23.5 (0.8)	1.49 (1.27–1.74)
College graduate	13.4 (0.6)	1 [Reference]
**Marital status**		
Married	19.1 (0.5)	1 [Reference]
Single	34.2 (1.3)	1.06 (0.88–1.28)
Divorced/separated/widowed	21.9 (0.8)	1.14 (0.98–1.32)
**Annual household income, $**		
<25,000	32.4 (1.2)	1.4^d^ (1.18–1.83)
25,000–49,999	25.8 (0.9)	1.3^d^ (1.11–1.61)
50,000–74,999	20.3 (1.1)	1.14 (0.94–1.38)
≥75,000	16.1 (0.8)	1 [Reference]
Don’t know/refused/missing	28.0 (1.7)	1.56 (1.21–2.00)
**Fruit intake**		
<1 time/d	29.5 (0.8)	1.37 (1.20–1.56)
≥1 time/d	18.4 (0.6)	1 [Reference]
**100% fruit juice intake**		
<1 time/d	23.5 (0.6)	0.73 (0.63–0.85)
≥1 time/d	25.0 (1.1)	1 [Reference]
**Vegetable intake**		
<3 times/d	24.9 (0.5)	1.13 (0.93–1.36)
≥3 times/d	17.5 (1.2)	1 [Reference]
**Leisure-time physical activity^d^**		
Highly active	20.8 (0.8)	1 [Reference]
Active	20.0 (1.1)	0.93 (0.78–1.10)
Insufficiently active	24.7 (1.2)	1.14 (0.95–1.36)
Inactive	30.0 (1.1)	1.47 (1.24–1.73)
**Smoking status**		
Nonsmokers	21.3 (0.7)	1 [Reference]
Former smokers	17.9 (0.8)	0.91 (0.79–1.05)
Current smokers	40.5 (1.4)	1.98 (1.68–2.33)
**Alcohol intake^e^**		
None	26.4 (0.8)	1 [Reference]
Any	22.0 (0.7)	0.81 (0.70–0.92)
Heavy	25.2 (1.7)	0.66 (0.53–0.84)
**Weight status^f^**		
Underweight	25.8 (3.6)	0.81 (0.49–1.34)
Normal weight	23.6 (0.9)	1 [Reference]
Overweight	23.0 (0.8)	1.02 (0.88–1.18)
Obese	26.2 (1.1)	1.10 (0.93–1.30)

Abbreviations: SE, standard error; OR, odds ratio; CI, confidence interval; BMI, body mass index.

a Sugar-sweetened beverages include nondiet soda and fruit drinks that are not 100% juice.

b Reference category included adults who did not drink sugar-sweetened beverages.

c Included 32,374 adults with data for all variables studied.

d Leisure-time physical activity was categorized as highly active (>300 min/week), active (150–300 min/wk), insufficiently active (11–149 min/wk), and inactive (no physical activity).

e Alcohol intake was categorized as none, any (≥1 drink of any alcoholic beverage during the past month but not heavy drinking), and heavy (>2 drinks/d for men and >1 drink/d for women).

f Underweight = BMI <18.5 kg/m^2^; normal weight = BMI of 18.5 kg/m^2^ to 24.9 kg/m^2^; overweight = BMI of 25.0 kg/m^2 ^to 29.9 kg/m^2^; obese = BMI ≥30 kg/m^2^.

## Discussion

Although only 2 types of SSBs (regular soda and fruit drinks) were included in our analyses, the prevalence of adults consuming any SSB per day was somewhat higher in our study (70%) compared with that reported in the NHANES 2005–2008 data, which showed that about 50% of adults aged 20 years or older consumed SSBs daily (3). In our study, 23.9% of adults consumed SSBs 1 or more times per day, with 10.7% doing so 2 or more times per day. This finding is similar to SSB intake reported for New York State adults, in which 20.5% consumed SSBs (regular soda, fruit drinks, sports drinks, or sweet tea) 1 or more times per day in the 2009 New York State BRFSS, which used 2 questions (25). In contrast, reported SSB intake was higher among Indiana adults in the 2011 Indiana BRFSS, which used 1 question; 64.9% Indiana adults drank SSBs (eg, soda, fruit drinks, sweet tea, sports drinks, energy drinks, coffee drinks) once or more per day, and 35.1% drank SSB twice or more per day (26). Discrepancies between studies could be due to variability in types of SSBs included in the questions used in BRFSS telephone surveys or variability in study samples because we pooled data from 6 states. The NHANES study used 24-hour diet recall for several types of SSB options, whereas the BRFSS is based on an abbreviated food-frequency question capturing only 2 types of SSBs to determine frequency of SSB consumption in the past month. Furthermore, we found that adults who lived in Delaware, Iowa, or Wisconsin had higher odds for drinking SSBs once or more a day than those who lived in Minnesota; however, the underlying reasons for these differences are unclear. Despite the variability of subgroup SSB consumption, the prevalence of SSB intake among US adults suggests a need for some adults to reduce their calorie intake from added sugars per the Dietary Guidelines for Americans 2010 (2). Thus, public health efforts to decrease SSB intake among high consumers of SSBs may be one strategy to reduce calorie consumption.

Similar to our findings, findings of previous studies showed that SSB intake was higher among younger adults, men, non-Hispanic blacks, adults with a less than high school education, and lower-income adults (3,17). We found that age was an important factor with the odds of consuming SSBs 1 or more times per day being more than 3 times higher among those aged 18 to 29 years than among those aged 70 years or older. One potential explanation for high SSB consumption may be lack of knowledge, because the sociodemographic characteristics of high SSB consumers in our study are somewhat similar to the characteristics of subpopulations who are not aware of calorie content of SSBs described in other studies. For example, 1 study reported that only 20% of adults knew the approximate calorie content of a 24-ounce soda, and the proportion of adults who knew the approximate calorie content of a 24-ounce soda was lowest among non-Hispanic blacks, those with a less than high school education, and those with lower household income (27). Another study conducted in the rural lower Mississippi Delta that did not specifically address SSB knowledge found that adults in the lowest health literacy category consumed 230 kilocalories (kcal) per day of SSBs, whereas those in the adequate health literacy category consumed 111 kcal per day of SSBs (28). To reduce disparities in SSB intake, targeted intervention efforts to reduce SSB intake among these high consumers may be needed.

To our knowledge, there is limited information on behavioral factors associated with SSB intake. We found that lower consumption of fruits and higher consumption of 100% fruit juice were significantly associated with greater odds for SSB intake at least once per day, but vegetable intake was not associated with daily SSB intake. Sharkey and colleagues reported that among rural adults, SSB intake was higher among those who consumed fewer than 5 servings per day of fruits and vegetables compared with those who consumed 5 or more servings per day (15). However, SSB intake was not significantly associated with fruit and vegetable intake among urban adults living in Texas (15). In this study, the odds for consuming SSBs 1 time or more per day were higher among physically inactive adults and current smokers. In contrast to our findings, Bermudez et al reported that SSB intake was not associated with physical activity and smoking status among US adults aged 20 to 39 years (19). Discrepancies between studies could be due to a difference in age of study populations or types of SSB included in the definition. For example, Bermudez et al included regular carbonated drinks or sodas, fruit drinks, and artificially flavored drinks, whereas we included regular soda and fruit drinks. In our study, alcohol drinkers had lower odds for consuming SSB daily compared with nondrinkers. It is possible that nondrinkers may choose to consume SSB instead of alcoholic beverages. Bleich and colleagues also found low consumption of alcohol among SSB consumers and reported that only 23% US adults who consumed at least one SSB per day drank alcohol (17).

Our study has several limitations. First, BRFSS information is self-reported, which could result in response bias in recall or social desirability. However, other studies have shown that estimates of beverage intake derived from responses to food-frequency questionnaires were similar to estimates derived from responses to 24-hour diet recalls or to food records (29,30). For example, a correlation coefficient between the food-frequency questionnaire and 24-hour diet recall for mean intake of SSB was 0.69 in a previous study (30). Second, only 2 types of SSBs (regular soda and fruit drinks) were available in the data set. However, these 2 SSBs represented the most commonly consumed types of SSBs among adults (16,17). Third, we are aware of 7 states (Alaska, California, Colorado, Connecticut, Indiana, New York, and Rhode Island) that used different SSB questions as State Added Questions in 2011. Because the questions were not the same, we were not able to include data from these additional states. We used a convenience sample, so our sample does not represent the entire US adult population, which limits the generalizability of our findings. Fourth, SSB consumption was surveyed in terms of frequency, so we cannot quantify associations between the volume of SSB consumption and the covariates of study. Fifth, about 10% of adults were excluded from the study because of missing SSB intake data, and the analytic sample had a slightly lower proportion of adults aged 70 years or older and a higher proportion of non-Hispanic whites. However, this exclusion would have minimal influence on the results, because SSB intake is higher among younger adults and lower among non-Hispanic whites based on findings of a previous study (3). Last, the associations are cross-sectional and do not permit assessment of causality or ascertaining the direction of the association.

In conclusion, about 1 in 4 adults consumed regular soda or fruit drinks at least once a day, and almost 1 in 9 did so at least twice daily. Additionally, our study showed that daily SSB intake varied by state and was notably higher among younger adults, men, non-Hispanic blacks, adults with lower education levels, and adults with lower household income or with missing income data. We also found increased odds of consuming SSBs 1 or more times per day among adults with fruit intake less than once a day, those who did not participate in leisure-time physical activity weekly, and current smokers; odds were lower among adults who consumed 100% fruit juice less than once a day and among alcohol drinkers. Various geographic, sociodemographic, and behavioral factors were associated with SSB intake in our study. States included in our analyses, in particular those with a higher prevalence of daily SSB intake, can use this data to guide their decisions about developing targeted interventions for improved nutrition as part of an obesity strategy, for example to decrease SSB intake and encourage the consumption of more healthful beverages (eg, plain water) among high-risk populations (eg, men, smokers). Other states could use these findings to educate potential high consumers of SSBs in their state about reducing SSB intake. Furthermore, states may consider using standardized SSB questions such as the BRFSS Optional Module to help monitor change over time. Where program evaluation is lacking, surveillance data can be helpful to evaluate community and state programs (eg, the Community Transformation Grant, Communities Putting Prevention to Work) and will allow comparisons of SSB intake across states.
